# The evidence of proteases in sprouted seeds and their application for animal protein digestion

**DOI:** 10.1007/s11696-017-0341-2

**Published:** 2017-12-04

**Authors:** Rafał I. Rawski, Przemysław T. Sanecki, Małgorzata Dżugan, Klaudia Kijowska

**Affiliations:** 1 Faculty of Biology and Agriculture, University of Rzeszow, 35-601, Rzeszow, Poland; 2 Faculty of Chemistry, Rzeszow University of Technology, 35-959, Rzeszow, Poland

**Keywords:** Sprout, Protease, Plant protein, Animal protein, Food technology

## Abstract

It has been experimentally proven that germinated plant seeds, such as leek, red clover, broccoli, and others contain proteases, which are able to digest animal protein such as gelatin, bovine albumin, casein, and egg albumin. Preliminary tests were carried out with the use of a semi-qualitative gelatin test, which is often applied to prove the presence of fruit enzymes. Quantitative examinations were carried out with the use of a ninhydrin reaction for amino acid determination as well as the Bradford and Smith methods for protein determination. Respective calibration curves were obtained for glycine (amino acid analysis) as well as for egg albumin, bovine albumin, and gelatin (protein analysis), with a full statistical evaluation including Mandel and Lack-of-Fit tests to check for linearity. It has been proven that the selected germinated seeds containing proteases can be applied as an effective aid for animal protein digestion.

## Introduction

The domination of processed food in the modern human diet results in a lack of active exogenous enzymes and as a consequence—a rapid depletion of their metabolic reserves. Since enzymes found in food are an important part of maintaining a healthy diet (Howell 1985; Urbano et al. 2005; Màrton et al. 2010a, b), it has become a common practice to supplement one’s diet with them (Roxas 2008). Furthermore, proteases such as bromelain and papain are used as a valid therapeutic agent in both modern and natural medicine (Maurer 2001; Roxas 2008; Tochi et al. 2008; Lopez-Otin and Bond 2008). The food rich in active enzymes, especially proteases, is applied as an important therapeutic and anti-aging diet component.

It is generally known that enzymes present in fruits such as pineapple, papaya, kiwifruit, and figs (bromelain, papain, actinidin, and ficin, respectively) are able to digest animal proteins, e.g., gelatin (Feijoo-Siota and Villa 2011; Ha et al. 2012; Boland 2013) or other hard to digest (“slow”) proteins such as casein (Boirie et al. 1997; Dangin et al. 2001). There are many commonly used qualitative tests using gelatin which can prove the presence of enzymes in the aforementioned fruits (Rutherfurd et al. 2011; Boland 2013; Kaur and Boland 2013). There is clear evidence that the green kiwifruit, and the enzyme actinidin itself, can enhance upper-tract digestion (gastric digestion in particular) of a variety of food proteins, which supports the use of kiwifruit as a dietary digestive aid (Rassam and Laing 2006; Boland 2013; Kaur and Boland 2013). These fruit enzymes are used also in the form of commercially available tablets.

The literature on seed germination states that the enzymes released during this process digest the seed’s own endogenous proteins (Adkins 1920; Mounfield 1936; Boulter and Barber 1963; Amen 1968; Shain and Mayer 1968; Rotari et al. 1997; Beilinson et al. 2002). The question is whether the plant enzymes present in the sprouted seeds are able to digest animal, i.e., foreign, protein effectively as well. There is no evidence on this subject in the literature, in contrast to the vast number of papers focused on the digestive application of the four fruit proteases (Rassam and Laing 2006; Roxas 2008; Rutherfurd et al. 2011; Boland 2013; Kaur and Boland 2013). The only trace is a paper by Jinka et al. (2009) where activity of sprout cysteine protease was measured with the use of azocasein as a reference protein. Information on the effectiveness of enzymes derived from germinated seeds can be significant for the preparation of various diets, industrial processing of animal proteins, designing prepared food rations, in digestive and absorption disorder therapy, and ultimately for the meat and dairy industries (Roxas 2008; Jinka et al. 2009). The search for new proteases, which can be used in more or less pure form in digestion supplements, seems to be reasonable. Sprout proteases, could be produced in moderate climate, probably at lower costs in contrast to classical proteases produced in tropics. The aim of this paper is to prove the factual presence of proteases in sprouted seeds, their ability to digest different animal proteins and indicate a possibility of their application.

## Experimental

### Reagents and laboratory equipment

#### Proteins

Egg albumin (Acros Organics, 400450500), bovine serum albumin (BioShop, ALB001.50, ≥ 98%), casein (Acros Organics, 276071000), and gelatin (POCH, 901946119, pure for analysis).

#### Standards used for calibration

Papain for biochemistry (Acros Organics, 416760100) and glycine (BioShop, GLN001.500, ≥ 99%).

#### Other reagents

Ninhydrin (Fluka Analytical, 33437-10G, ≥ 99%), acetone (pure for analysis), ethanol (pure for analysis), Triton X-100 (Windsor Laboratories Limited, pure for analysis), Coomassie Brilliant Blue (Sigma-Aldrich, G-250, 100%), phosphoric acid (CHEMPUR, pure for analysis), bicinchoninic acid disodium salt hydrate (Sigma-Aldrich, D8284-1G, ≥ 98%), sodium carbonate (anhydrous, CHEMPUR, pure for analysis), sodium hydrogen carbonate (CHEMPUR, pure for analysis), tartaric acid (POCH, pure for analysis), and sodium hydroxide (POCH, pure for analysis).

#### Laboratory equipment

Spectrophotometer (Hitachi U-1900, Japan), automatic sprouter (EasyGreen MicroFarm, USA), centrifuge (MPW 56, Poland), sand bath, and other standard laboratory equipment.

### Seed germination

The commercially available organic seeds were sterilized in 0.1% H_2_O_2_ for 1 min, then drained and washed three times in distilled water. After that, the seeds were placed into germination trays and germinated at 20–25 °C in an Easy-Green Microfarm automatic sprouter, previously sterilized with the use of 10% H_2_O_2_. The seeds were automatically rinsed four times a day with distilled water and illuminated for 10 h each day. The sprouted seeds were considered to be ready for investigation either when the protruding sprout length was equal to the parent seed’s diameter, or when the time of sprouting was considered to be optimal (2–5 days, depending on sprout kind). The following seeds were used: leek (*Allium ampeloprasum* L.), broccoli (*Brassica oleracea* L.), red clover (*Trifolium pratense* L.), red cabbage (*Brassica oleracea* var. *capitata* f. *rubra*), sunflower (*Helianthus annuus* L.), mung bean (*Vigna radiata* L.), fennel (*Foeniculum vulgare*), black mustard (*Brassica nigra* L.), carrot (*Daucus carota* subsp. *sativus*), and radish (*Raphanus sativus* L.).

### Sample preparation

#### Sprouted seed extract sample

Five grams of sprouted seeds were homogenized with 100 ml of distilled water using a rotary blade blender for 3 min with 10 s breaks to avoid overheating. After 2 h of incubation at 20 °C, 10 ml of decanted solution (pH ≈ 7) was poured into a 15-ml falcon test tube and centrifuged (15 min, 6000 rpm, 2214×*g*, fixed angle MPW rotor no. 11140, MPW 56 centrifuge) with the addition of 1 μl 1% non ionic surfactant Triton X-100. The supernatant was tested immediately.

#### Sprouted seed extract with protein sample

This sample was made the same way as the sprouted seed extract sample, with the difference that 100 ml of protein solution (4 mg/ml, pH ≈ 7) was added instead of 100 ml of pure water at the beginning of the preparation process.

#### Protein solution

Protein samples were prepared from pure protein and twice-distilled water to obtain the required concentration: 4 mg/ml for egg albumin, bovine albumin, and gelatin; 1 mg/ml for casein.

### Sample analysis

All samples were, after 2 h of incubation at 20 °C, immediately examined for amino acid concentration with the ninhydrin method (Moore and Stein 1948) and for protein concentration with both the Bradford (Bradford 1976) and Smith (Smith et al. 1985) methods. Contrary to standard procedure, the complete VIS adsorption curves were recorded (Hitachi U-1900) to fully monitor the process and detect possible disturbances such as turbidity. All tests were repeated five times.

### Calibration

All regression models were determined using data from three independent measurements via Origin software and tested with the use of Lack-of-Fit, Fisher—Snedecor, IUPAC, and Mandel tests to verify the correctness of the model used (Rawski et al. 2016).

#### Ninhydrin test

Pure glycine was used for the Ninhydrin test calibration. The linear model, *y* = *ax* + *b*, for concentration range $0 - 0.014 \; \frac{{\mu {\text{g}}}}{\text{ml}}$ and eight calibration points, was determined as Eq. (1), where Δ*a* and Δ*b* are respective standard deviations (Rawski et al. 2016).

1 $$y = 83.70x + 5.1553 \cdot 10^{-4};\; \Delta a = 0.9476;\; \Delta b = 8.601 \cdot 10^{-3} ;\; r^{2} = 0.9999$$

#### Bradford method

Egg albumin was used for the Bradford method calibration. The quadratic model, $y = ax^{2} + bx + c$, for $0 - 20 \; \frac{{\mu {\text{g}}}}{\text{ml}}$ protein concentration range was determined for 11 calibration points as Eq. (2), where Δ*a*, Δ*b* and Δ*c* are respective standard deviations (Rawski et al. 2016).

2 $$y = - 5.938 \cdot 10^{-4} x^{2} + 0.03375x + 0.01155;\;\Delta a = 2.182 \cdot 10^{-4} ;\; \Delta b = 4.532 \cdot 10^{-3} ;\;\Delta c = 0.01947;\; r^{2} = 0.9950$$

#### Smith method

Both bovine albumin and gelatin were used for the Smith method calibration. The quadratic model, $y = ax^{2} + bx + c$, for bovine albumin concentration range $0 - 2000 \; \frac{{\mu {\text{g}}}}{\text{ml}}$ and nine calibration points, was determined as Eq. (3), where Δ*a*, Δ*b* and Δ*c* are respective standard deviations (Rawski et al. 2016).

3 $$y = -2.316 \cdot 10^{-7} x^{2} + 0.001560x + 0.02125;\;\Delta a = 7.765 \cdot 10^{-8} ;\; \Delta b = 1.521 \cdot 10^{-4} ;\; \Delta c = 0.05094;\; r^{2} = 0.9982$$

The quadratic model for gelatin concentration range $0 - 4000 \; \frac{{\mu {\text{g}}}}{\text{ml}}$ and 10 calibration points was determined as Eq. (4).

4 $$y = - 5.561 \cdot 10^{ - 8} x^{2} + 5.958 \cdot 10^{ - 4} x + 0.04780;\;\Delta a = 2.886 \cdot 10^{ - 8} ;\; \Delta b = 1.152 \cdot 10^{ - 4} ;\; \Delta c = 0.06946; \;r^{2} = 0.9883$$

## Results and discussion

### Semi-quantitative gelatin test

**Fig. 1:**
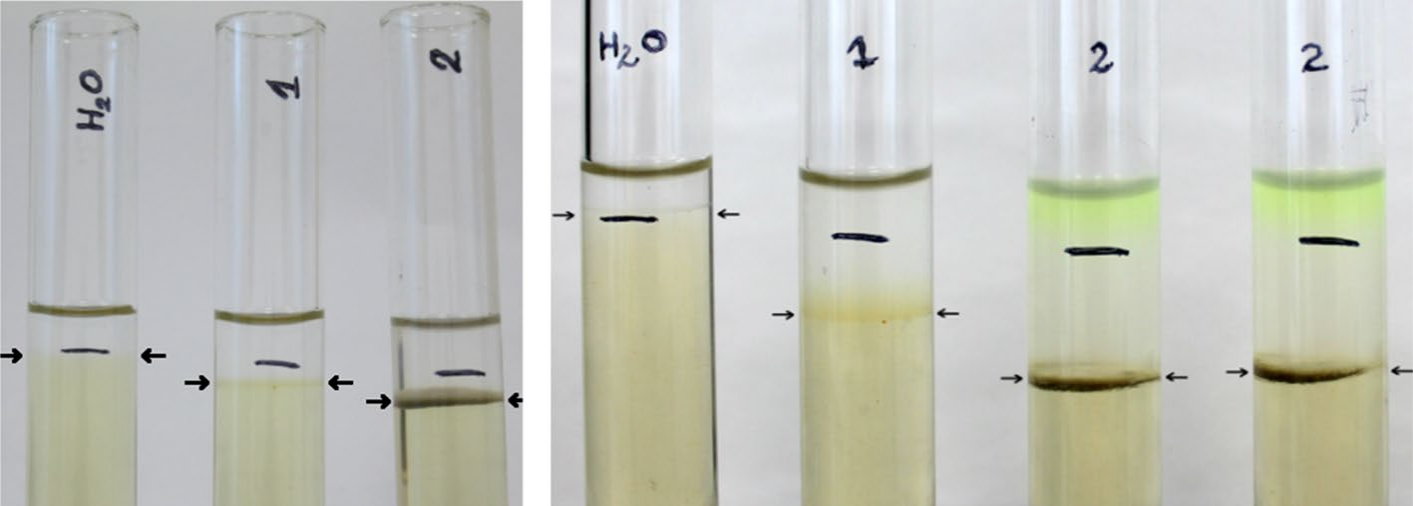
Solid gelatin tube test for sprout enzyme activity measurement and comparison with fruit papain. Samples: distilled water (H_2_O), papain (1), and leek (2). Duration of the experiment: 4 h (left picture) and 24 h (right picture); temperature: 25 °C; gelatin concentration: 4 g/100 ml; sprout extract concentration: 5 g/10 ml; papain concentration: 0.12 g/100 ml. Black line indicates the initial level of solid gelatin. The results obtained from repeated experiments were the same within ± 0.5 mm

To compare the proteolytic activity of the tested sprouted seeds with the well-known enzymatic activity of fruit enzymes, a gelatin test was done. It involved placing identical amounts of sprout extract, pure papain standard (0.12 g/100 ml, positive sample), and water (negative sample) into separate test tubes, each of which contained the same amount of solid gelatin. The proteolytic activity was measured as the depth of gelatin penetration (fluidization). Figure 1 demonstrates the enzymatic strength of the sprouted seed extracts in gelatin degradation and makes it possible to compare it with standard enzymes. An increase of sprout extract concentration from 5 g/100 ml to 5 g/10 ml resulted in deeper gelatin fluidization.

Despite its simplicity, the solid gelatin tube test can be considered to be a semi-quantitative version of a common qualitative gelatin test. It makes it possible to compare the activity of various enzymes in relation to gelatin protein degradation. According to the data from Fig. 1, it enables a simple comparison of commercial and sprout enzymes, and a semi-quantitative comparison of the enzymatic activity of various sprout species extracts.

### Comparison of various sprout protease activity using the Ninhydrin and Bradford methods

To select the seeds with the most active proteolytic enzymes, ten sprout species were tested five times each for an amino acid concentration increase with the Ninhydrin method and for a protein concentration decrease with the Bradford method. The ninhydrin test showed the highest activity of leek, red clover, and broccoli sprouted seeds. Although the Bradford test showed better results for the alfalfa, mung bean, and sunflower sprouted seeds, those results were discarded due to high sample turbidity. Without those results, the Bradford test confirmed the results obtained from the ninhydrin test even though its absorbance range was overflowed Eq. (2). Therefore, the leek, red clover, and broccoli seeds were selected for further tests.

### Determination of the optimal sprouting time

**Fig. 2:**
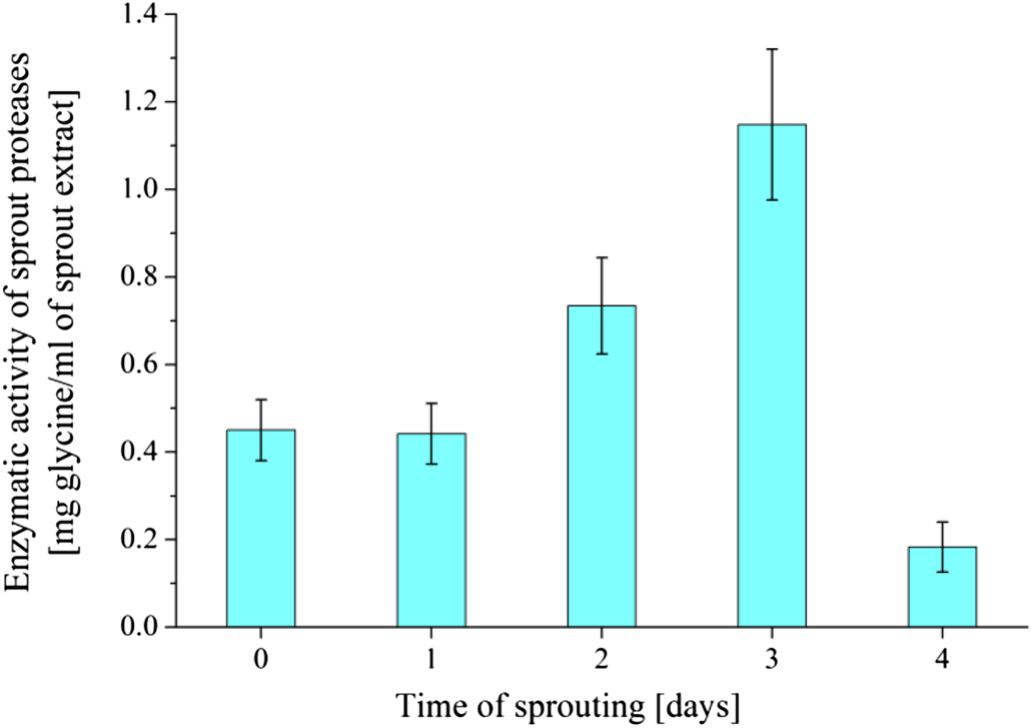
Enzymatic activity of proteolytic enzymes vs. time of germination for sprouted broccoli seeds measured by ninhydrin method. The “0 day” of sprouting shows the amount of residual free amino acids present in dry seeds and in the animal protein. Confidence intervals, calculated at* t*_0.1; n-1_, are shown as whiskers on the respective bars

To achieve sprouted seeds that possess the highest enzymatic activity, a determination of the optimal time of sprouting was an important step. During seed germination process lasting 4 days, after each day, 5 g sample of sprouted seeds was tested by ninhydrin method (Fig. 2) against egg albumin. The data for broccoli seeds are presented as an example below.

Ninhydrin method shows that for broccoli seeds the highest activity of sprout peptidases can be obtained after 3 days of germination. As a result, 3 days were set as a standard time of broccoli seed germination. The optimal time of sprouting, obtained by presented method, was 2–5 days, depending on seed kind. For leek seeds, more widely studied here, the respective time was 3 days.

### Comparative casein digestion weight test

To quantitatively compare the activity of sprout proteases with a commercial protease, a casein weight test was performed. 0.3 g sample of casein was mixed with 5 cm^3^ of sprout extract or 5 cm^3^ of papain standard solution (1.5 g/dm^3^). After 1 day, the remaining casein was washed with water, and dried under vacuum. The remaining casein was weighed and the mass loss was determined. Possible casein swelling effect has been eliminated by parallel measurement of the reference sample with sprout extract deactivated by boiling or with pure water instead of papain (Table 1). Mass loss was calculated as a difference between mass of casein reference sample and its mass from investigated sample. The results are shown in Table 1.

**Table 1:** The results of comparative weight test of leek protease and papain activity against casein protein

Investigated sample / reference sample	Weight decrease (%)
Fresh leek extract 5 cm^3^, casein 0.3 g / deactivated leek extract 5 cm^3^, casein 0.3 g	17 ± 2
Papain standard solution 5 cm^3^, casein 0.3 g / water 5 cm^3^, casein 0.3 g	30 ± 2

The results of casein weight test demonstrate that the leek sprout extract, exhibits about 57% of the purified papain activity. Knowing that the papain used as a reference has an activity of 46,000 U/mg (BIOZYM GE mbH), one can estimate that the proteases found in leek sprout extract exhibit an activity of about 26,220 U/mg.

### Boiling test of leek sprouted seeds

**Fig. 3:**
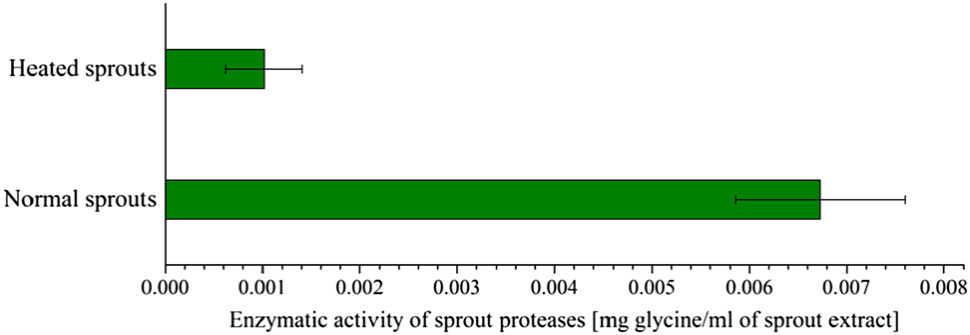
Comparison of the proteolytic activity against gelatin of boiled and fresh leek sprout samples within a 2-h time distance. Sprouted seeds were heated at 100 °C for 1 h, amino acid concentration was determined with the use of the ninhydrin method. The experiment was repeated five times; $\%_{\text{error}} \le 10\%$. Confidence intervals, calculated at *t*_0.1.*n−*1_, are shown as whiskers on the respective bars

To show that the proteases are solely responsible for the degradation process and that there are no other substances in the sprout extract sample that can produce such digestive effect, a heat treatment test with the use of the Ninhydrin method was carried out. The results of the test clearly indicate that there is a significant difference between results obtained for boiled and non-boiled samples (Fig. 3). After boiling, the sprout proteases lost most of their activity. Thus, this test proves that the sprout proteolytic enzymes are the main factor responsible for the animal protein degradation.

### Quantitative tests

#### Bradford method

**Fig. 4:**
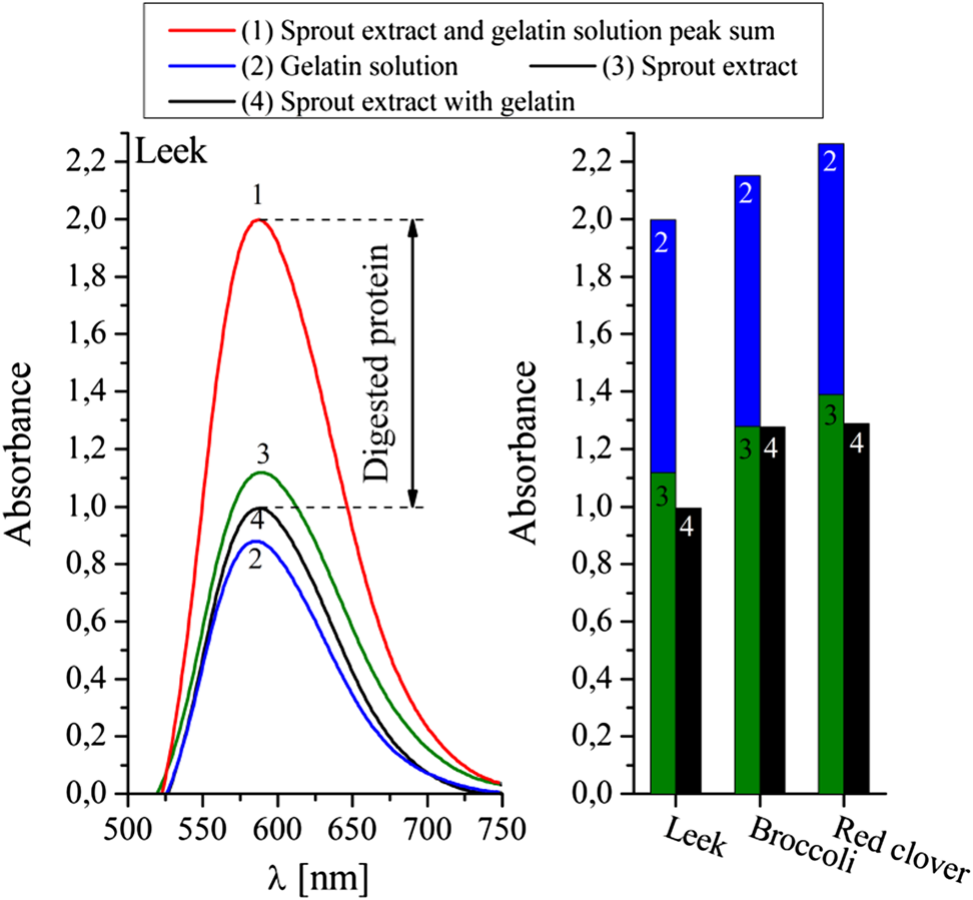
The activity of proteases found in sprouted seeds of leek, broccoli, and red clover shown as a difference between total protein concentration at zero (1, red) and after 2 h of reaction (4, black). Total protein concentration at zero time was calculated as the sum of gelatin solution (2, blue) and sprout extract (3, green) samples. All results were obtained via the Bradford method; the left image shows the original spectrum, the right image represents the maximum peak absorbance from respective spectra. The experiment was repeated five times; $\%_{\text{error}} \le 10\%$

The sprouted seeds of leek, broccoli, and red clover were tested five times with the Bradford method, using gelatin as an external protein. Three types of samples were used: sprout extract with gelatin (post-reaction sample), sprout extract sample, and pure gelatin sample. Both the sprout extract and the pure gelatin sample runs were combined to create an initial protein concentration sample. The difference between the post-reaction sample run and the initial protein concentration sample run was measured as the protein content decrease. The results are shown in Fig. 4.

In Fig. 4, there is a clear difference between the initial protein concentration (1, red) and post-reaction (4, black) samples. This indicates that the amount of protein has diminished significantly. Therefore, it can be stated that all of the tested sprouted seeds contain very active proteolytic enzymes. Unfortunately, due to the fact that the absorbance results overflow the range of the Bradford method’s calibration curve Eq. (2), they cannot be converted into protein concentration and can only be considered as half-quantitative.

However, these results are good enough to show an interesting feature of sprout proteases. In Fig. 4, the difference between the initial protein concentration (1, red) and the post-reaction (4, black) samples is far greater than between the post-reaction (4, black) and the sprout extract (3, green) samples. This indicates that the native sprout protein vanishes considerably slower than the external animal protein. Therefore, sprout proteases are probably more active towards external proteins than towards their own ones. Unlike in the external protein, there must be a mechanism built into the endosperm’s protein structure which controls the digestion rate of the native protein.

#### Ninhydrin method

To support the results of the Bradford method, a test using the ninhydrin method was done. The activity of sprout proteases from leek, broccoli, and red clover was tested five times against gelatin, bovine albumin, and casein.

**Fig. 5:**
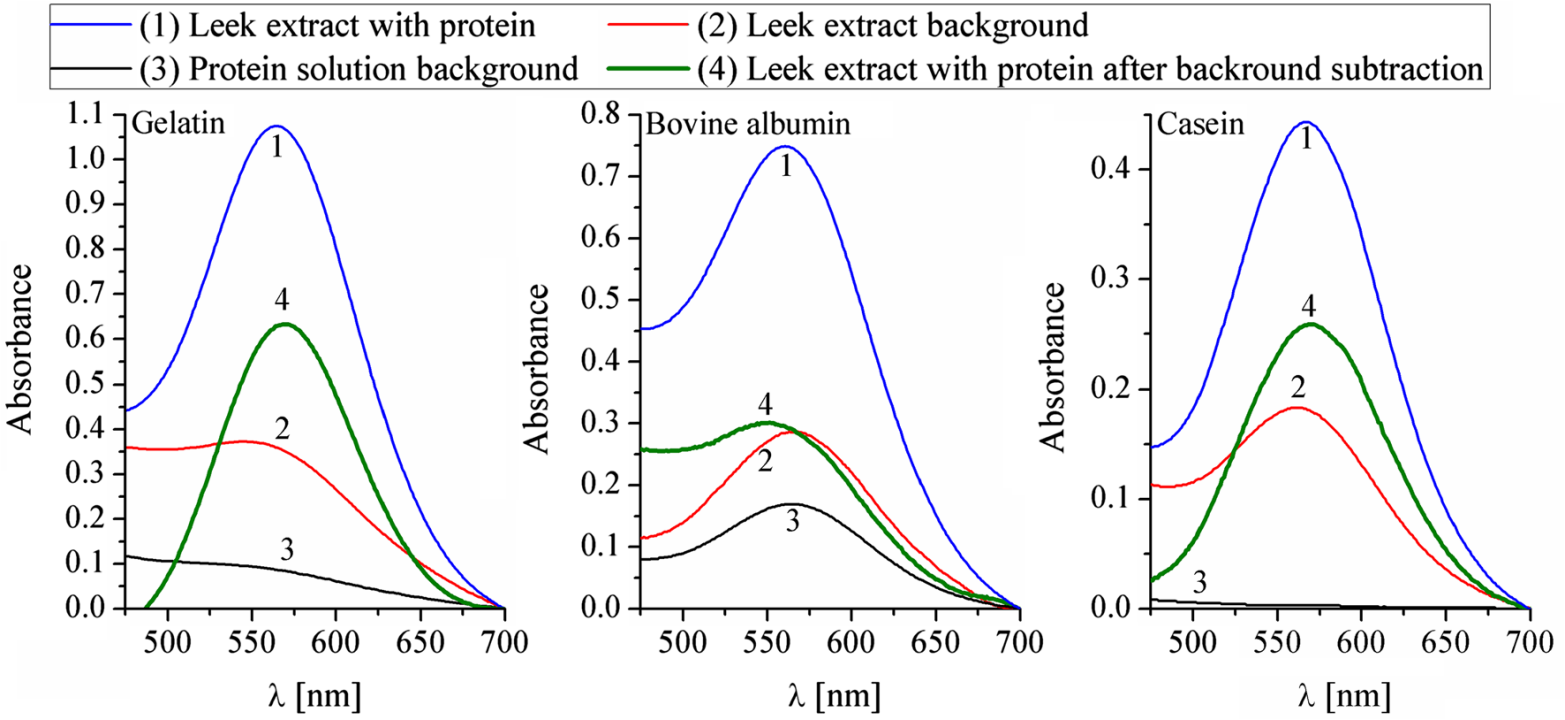
The efficiency of protein digestion by a leek sprout’s proteolytic enzymes, as measured with the ninhydrin method. All of the obtained results fit into the linear calibration model of the method Eq. (1) and, therefore, can be considered as quantitative and easily recalculated into glycine concentration. Repeated five times; $\%_{\text{error}} \le 10\%$

**Fig. 6:**
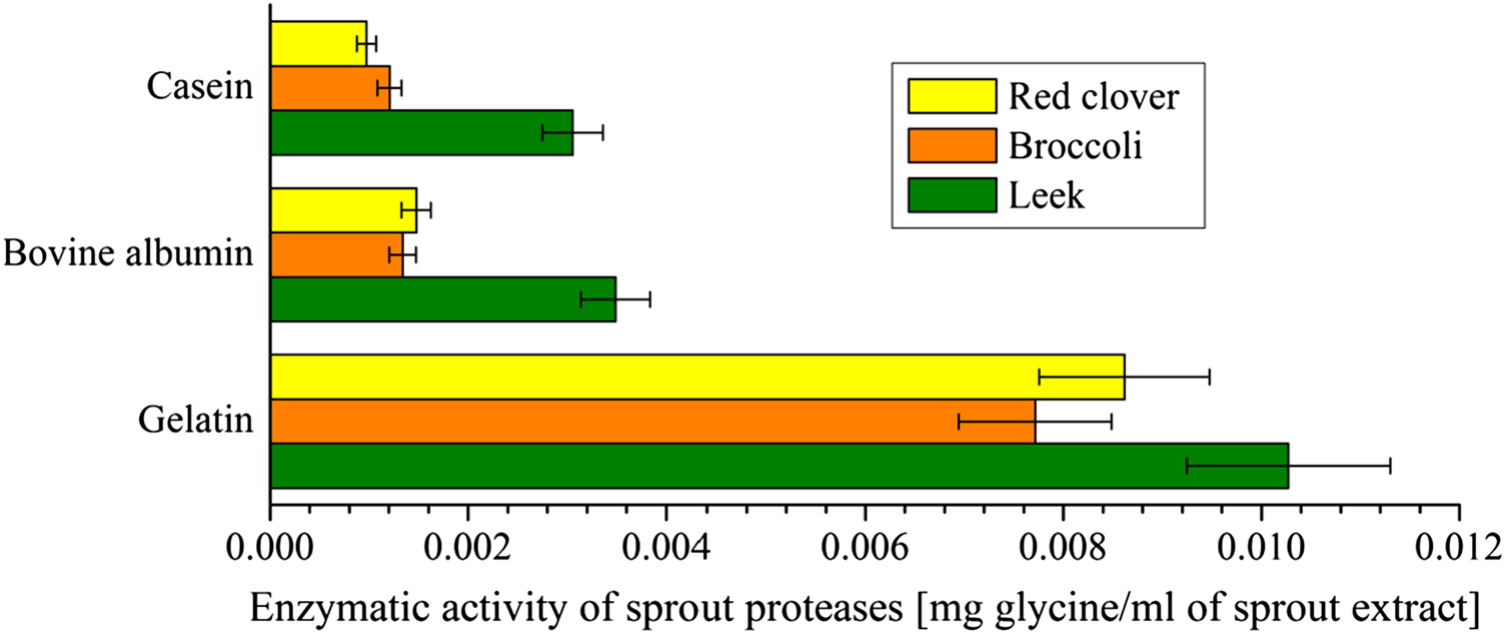
Efficiency of protein digestion by proteolytic enzymes of the three sprout species. Ninhydrin method. The bars represent absorbance values from Fig. 5, recalculated using the linear calibration model for the ninhydrin method Eq. (1). The experiment was repeated five times; $\%_{\text{error}} \le 10\%$. Confidence intervals, calculated at *t*_0.1,*n−*1_, are shown as whiskers on the respective bars

The ninhydrin method involved the same three types of samples as the Bradford method, although further data treatment was different. Both the reference and blank sample runs were subtracted from the post-reaction sample as background. The result, indicated by a green (4) line in Fig. 5, was the measure of amino acid concentration created by animal protein digestion. The effects of external protein degradation by germinated seeds are shown in Fig. 6.

The obtained confidence intervals, shown in Fig. 6 as whiskers, prove that the difference between leek sprouted seeds and the rest of the sprouted seeds is statistically significant for casein and bovine albumin. At the same time, the differences between red clover and broccoli data for casein and bovine albumin as well as the differences for all three gelatin bars are not statistically significant and those bars should be treated as having the same length within calculated confidence intervals. From Fig. 6, it is clear that leek proteases present the highest activity from among all the tested sprouted seeds. The difference is more evident for the hard-to-digest proteins (Boirie et al. 1997; Dangin et al. 2001): casein and bovine albumin. The ninhydrin method makes it possible to state, similarly as with the Bradford method, that sprout proteases are unquestionably effective against external proteins and can even digest resistant proteins fairly well.

#### Smith method

**Fig. 7:**
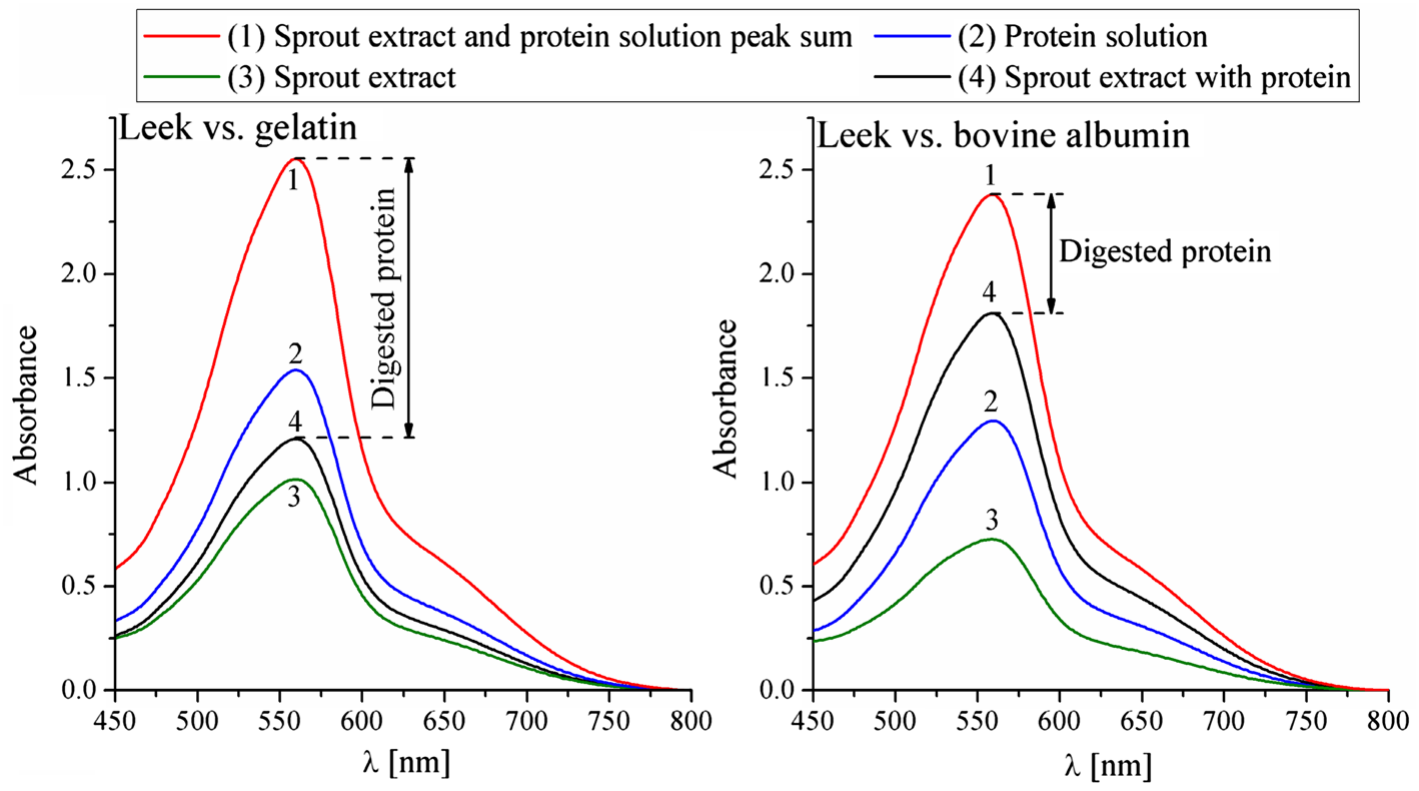
The activity of leek sprout proteases against gelatin and bovine albumin shown as a protein concentration difference between post-reaction (line 4, black) and complete reference (line 1, red) samples after 2 h. Smith method. The experiment was repeated five times; $\%_{\text{error}} \le 10\%$

To prove the conclusion derived from the Bradford method about sprout proteases’ higher activity towards external proteins than towards their own proteins as well as to confirm the results obtained from the ninhydrin method, the Smith method was used. The sample configuration and data treatment were the same as in the Bradford method as shown in Fig. 7. The experiment was repeated five times.

The results of the Smith method confirm the Bradford method’s conclusion about sprout enzymes’ higher activity towards external protein. The post-reaction sample (Fig. 7, line 4, black) and sprout extract sample plotlines (line 3, green) not being as close to each other as in the Bradford method’s results can be explained by the fact that the Smith method has a wider range of calibration models and is, therefore, more accurate. All results obtained with the use of the Smith method are within the range of its calibration model Eqs. (3) and (4), and can be converted into protein concentration.

**Fig. 8:**
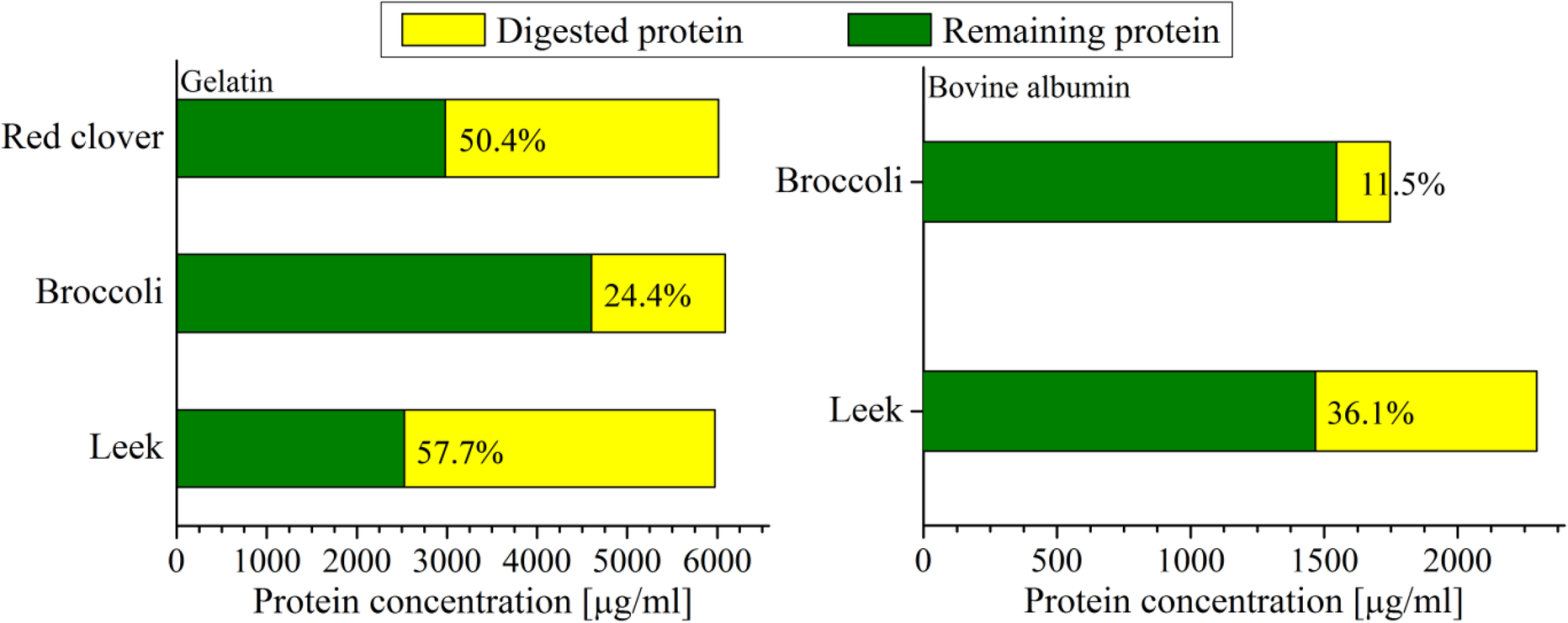
The efficiency of the selected types of sprouted seeds in relation to the digestion of gelatin and bovine albumin. Total bar height represents the amount of protein before digestion, the yellow color indicates the amount of protein digested enzymatically, and the green color indicates the protein that was left after the process. Smith method. The experiment was repeated five times. $\%_{\text{error}} \le 10\%$

The presented results (Fig. 8, Table 2) indicate that sprouted seeds in general can be applied as a digestion-supporting agent. The quantitative results once more confirm the exceptional value of leek sprouted seeds, as they are able to digest more than 50% of gelatin and more than thirty percent of bovine albumin. The results of the ninhydrin method also support the role of leek sprouted seeds as a digestion-supporting agent in relation to casein protein (Fig. 6). The red clover and broccoli sprouted seeds were found to be less active (Table 2, Fig. 8). However, due to their other valuable elements, such as sulforaphane in broccoli, it is a great way to include proteolytic enzymes in everyday meals.

**Table 2:** The efficiency of the three types of sprouted seeds in relation to the digestion of animal proteins: gelatin and bovine albumin

Sprout	Protein	Protein concentration (μg/ml)		Protein concentration decrease	
		0 h	2 h	(μg/ml)	(%)
Leek	Gelatin	5975.3	2525.3	3450.0	57.7
Leek	Albumin	2295.8	1466.6	829.18	36.1
Broccoli	Gelatin	6086.6	4603.3	1483.4	24.4
Broccoli	Albumin	1747.3	1546.6	200.70	11.5
Red clover	Gelatin	6012.3	2980.0	3032.3	50.4

Smith method. The experiment was repeated five times; $\%_{\text{error}} \le 10\%$

In summary, the results of all conducted tests indicate that the germinated leek seeds contain proteases that express competitive activity to commercially used proteases such as papain. The remaining test species, namely, broccoli and red clover, also contain active proteases, but their activity is not competitive with that of commercial papain. In conclusion, germinated leek seeds can be a good source of proteases which, after purification, can be an alternative to fruit proteases. However, the respective method of isolation and purification of the sprout proteases, as well as the question whether the sprout proteases remain active in the stomach’s acidic environment, remain outside the scope of this paper.

## Conclusions

1. The presence of active proteolytic enzymes in germinated seeds, able to digest external animal proteins, was proven with the use of three independent analytical methods for both amino acid and protein determination, as well as a gelatin test. The results were convergent with each other.
2. Despite the kinetic methods and kind of tests applied, the leek proteolytic enzymes were the most effective against gelatin, bovine albumin, and casein. The red clover and broccoli sprout enzymes were slightly less effective, with the largest differences visible in casein digestion.
3. Sprout enzymes, especially those found in sprouted leek seeds, are comparable with the commercially available fruit proteases.
4. For the investigation presented in this paper, the Smith method is more convenient than the Bradford method, as it allows operation in a wide protein concentration range even though its output spectral response is less regular and less defined.
